# A novel *NTRK1* splice site variant causing congenital insensitivity to pain with anhidrosis in a Chinese family

**DOI:** 10.3389/fgene.2024.1345081

**Published:** 2024-05-10

**Authors:** Ling Sun, Jin Dai, Yuan Zhang, Lijun Zhou, Yaqiong Ren, Hongying Wang

**Affiliations:** ^1^ Department of Cardiology, Children’s Hospital of Soochow University, Suzhou, Jiangsu, China; ^2^ Department of Orthopaedics, Suzhou, Jiangsu, China; ^3^ Department of Clinical Laboratory, Suzhou, Jiangsu, China; ^4^ Laboratory of Pediatric Research, Suzhou, Jiangsu, China; ^5^ Institute of Pediatric Research, Children’s Hospital of Soochow University, Suzhou, Jiangsu, China

**Keywords:** CIPA, NTRK1, minigene assay, mRNA splicing, novel splice mutation

## Abstract

**Background:**

Congenital insensitivity to pain with anhidrosis (CIPA, OMIM #256800), also known as hereditary sensory and autonomic neuropathy type Ⅳ (HSAN-IV), is a rare autosomal recessive disorder characterized by recurrent episodic fevers, anhidrosis, insensitivity to noxious stimuli, self-mutilating behavior and intellectual disability. CIPA can be caused by the variants in *NTRK1* gene, which encodes a high-affinity tyrosine kinase receptor for nerve growth factor. To ascertain the hereditary cause of a patient with CIPA accompanied by the additional symptoms of mild growth retardation, prone to fracture, underdeveloped nails of fingers and toes, irregular tooth alignment, enamel hypoplasia, postoperative wound healing difficulty, hand and limb deformity, and dislocation of hip joint, whole exome sequencing was used and revealed a compound heterozygous variant in *NTRK1*.

**Methods:**

DNA was extracted from peripheral blood samples of pediatric patients and their parents, and subjected to comprehensive analysis using whole-exome sequencing (WES), followed by verification of variant sites in the *NTRK1* gene through Sanger sequencing. To elucidate the functional impact of the newly discovered variants, an *in vitro* experimental system was established. Splicing analysis was conducted using PCR and Sanger sequencing, while expression levels were assessed through qPCR and Western blot techniques.

**Results:**

One hotspot variant c.851-33T>A(ClinVar ID: 21308) and a novel variant c.850 + 5G>A(ClinVar ID:3069176) was inherited from her father and mother, respectively, identified in the affected individuals. The c.850 + 5G>A variant in *NTRK1* resulted in two forms of aberrant mRNA splicing: 13bp deletion (c.838_850del13, p. Val280Ser fs180) and 25bp deletion (826_850del25, p. Val276Ser fs180) in exon 7, both leading to a translational termination at a premature stop codon and forming a C-terminal truncated protein. The expression of two abnormal splicing isoforms was decreased both in the level of mRNA and protein.

**Conclusion:**

In conclusion, this study elucidated the genetic cause of a patient with CIPA and identified a novel variant c.850 + 5G>A in *NTRK1*, which broadened the and enriched the *NTRK1* mutation spectrum.

## 1 Introduction

Congenital insensitivity to pain with anhidrosis (CIPA, OMIM #256800), also known as hereditary sensory and autonomic neuropathy type IV (HSAN-IV), is a rare autosomal recessive genetic disorder with an estimated prevalence of 1/125,000,000 and approximately 1/600,000-950,000 in Japan (Haga et al., 2013; Amin et al., 2017; Wang et al., 2018). CIPA primarily affects infants and children, with rare cases reported in adults. Due to the similarity of symptoms with other conditions such as hypohidrotic ectodermal dysplasia and osteogenesis imperfecta, CIPA was often misdiagnosed in clinical settings (Zhao et al., 2020). All individuals with CIPA exhibited two key features: insensitivity to pain and lack of sweating. As a result of impaired sweat gland function, CIPA patients experienced recurrent episodes of hyperthermia. Additionally, after the eruption of primary teeth, CIPA children might engage in self-injurious behaviors. Some patients also presented with intellectual disability and multiple fractures, which aided in confirming the diagnosis (Franco et al., 2016; Nam et al., 2017; Wang et al., 2017). The term "CIPA" was officially coined by Swanson in 1963 and had been widely used since then (Swanson, 1963). In 1975, Dyck and colleagues categorized HSAN into four types, with type I being autosomal dominant and types II, III, and IV being autosomal recessive. Type IV corresponds to congenital insensitivity to pain (Dyck et al., 1983).

In 1996, Indo et al., conducted a study on three unrelated CIPA patients and identified variants in the *NTRK1* gene (neurotrophic tyrosine kinase receptor 1) encoding the tyrosine kinase receptor. These variants led to the deletion of the tyrosine kinase domain. Mice lacking *Ntrk1* displayed similar symptoms of pain insensitivity to CIPA patients (Indo et al., 1996). The majority of CIPA cases were caused by biallelic variants in *NTRK1* (Yang et al., 2021). *NTRK1* (NM_001012331.1) is located on chromosome 1q21-22 and consists of 17 exons that encode a tyrosine kinase receptor called tropomyosin receptor kinase A (TrkA). TrkA is a transmembrane protein with an intracellular tyrosine kinase domain (encoded by exons 13-17), a single-pass transmembrane hydrophobic alpha-helical domain (encoded by exons 9-12), and an extracellular domain encoded by exons 1-8. The extracellular domain includes an internal signal peptide domain, two cysteine-rich domains, two immunoglobulin-like domains, and an LRR domain (Nam et al., 2017). TrkA is a high-affinity receptor for nerve growth factor (NGF). Upon NGF binding, TrkA forms dimers and activates the tyrosine kinase domain within the intracellular region, leading to autophosphorylation and activation of downstream signaling pathways such as mitogen-activated protein kinase (MAPK) and phosphatidylinositol 3-kinase (PI3K), which are involved in neuronal development and function (Ji et al., 2002; Shaikh et al., 2017). In addition to its role in promoting neuronal growth and development, NGF also sensitizes receptors on nociceptive neurons involved in temperature and pain perception (Indo, 2018). Variants in *NTRK1* result in the selective loss of NGF-dependent primary afferent neurons and postganglionic sympathetic neurons, leading to the insensitivity to pain and lack of sweating observed in CIPA patients (Indo, 2018).

To date, over 130 pathogenic variants in *NTRK1* gene have been reported, including splice site variants, missense variants, and frameshift variants, predominantly occurring in the extracellular and intracellular domains of the TrkA protein, with only a few occurring in the transmembrane region (Geng et al., 2018; Li S. et al., 2021). Moreover, several recurrent pathogenic variants have been identified in specific populations. For example, the Phe284Trpfs*36, c.1660del (Arg554Glyfs*104; ClinVar ID:21305), and c.2020G>T(p.Asp674Tyr; ClinVar ID:21307) variants occur in approximately 70% of Japanese patients, the c.1860_1861insT (Pro621Serfs*12; ClinVar ID:21305) variant occurs in almost 90% of Israeli patients, and the c.287+2dupT(ClinVar ID: 859211) pathogenic variant has been found in Chinese and Korean patients (Echaniz-Laguna et al., 2021).

Although there has been considerable research on the genetic etiology of CIPA, further studies are needed to understand the impact of novel variations on the *NTRK1* molecular structure and protein function, as well as the disease characteristics and severity in CIPA patients.

In this study, we performed whole exome sequencing (WES) analysis to investigate the genetic etiology of CIPA in the affected child and identified c.851-33T>A(ClinVar ID: 21308) mutation and a novel c.850 + 5G>A(ClinVar ID:3069176) variant in *NTRK1*. Additionally, we utilized computational prediction tools and conducted minigene experiments as well as *in vitro* expression assays to assess the impact of novel variations on the CIPA phenotype. Our findings expanded the spectrum of *NTRK1* variants and laid the groundwork for further functional studies of *NTRK1*. Moreover, our results provide valuable insights for the genetic diagnosis of CIPA patients.

## 2 Materials and methods

### 2.1 Subjects

A 7-year and 8-month-old girl, with a history of multiple fractures over the past 2 years, was admitted to our hospital due to lower limb deformity after fracture surgery more than 1 year ago. Despite self-reports and physical examinations, the cause could not be determined. Subsequently, the proband underwent whole-exome sequencing analysis.

### 2.2 Whole exome sequencing

Peripheral blood samples (2-4 mL) were collected from the patient and her parents. DNA extraction and WES analysis were performed by Berry Genomics (Beijing, China). WES was conducted using the Illumina NovaSeq 6000 platform (San Diego, CA) with PE150 mode, ensuring a sequencing depth of over 20× for more than 99.5% of the target regions. After obtaining raw data, quality control was conducted to remove reads that did not meet the requirements. The Burrows-Wheeler Aligner (BWA) software was used for alignment against the human reference genome sequence (hg19/GRCh37) available at the UCSC Genome Browser (https://genome.ucsc.edu/). Variant calling was performed using the Verita Trekker^®^ Variants Detection System (Berry Genomics) (Wang et al., 2010) and GATK v3.70. Annotation of variants was carried out using databases and tools such as RefSeq, dbSNP, 1,000 Genomes, ExAC, gnomAD, and the Enliven^®^ Variants Annotation System (Berry Genomics). Pathogenicity of variants and interpretation of data followed the guidelines of the American College of Medical Genetics and Genomics (ACMG) (Richards et al., 2015).

### 2.3 *In silico* analysis

For predicting the impact of this mutation on splicing at the mRNA level. The pathogenicity of missense variants was predicted using three algorithms: SpliceAI (https://spliceailookup.broadinstitute.org/), Varseak (https://varseak.bio/index.php), and the CBS algorithm.

### 2.4 Minigene splicing assay-construction of recombinant vectors

Two sets of recombinant vectors were constructed: pcMINI-*NTRK1*-wt/mut and pcDNA3.1-*NTRK1*-wt/mut. The minigene fragments inserted in the pcMINI-*NTRK1*-wt/mut vectors consisted of Intron6 (223bp)-Exon7 (133bp)-partial Intron7 (477bp). The minigene fragments inserted in the pcDNA3.1-*NTRK1*-wt/mut vectors consisted of Exon7 (133bp)-Intron7 (1877bp)-Exon8 (327bp). The construction process involved designing two pairs of nest primers: 10003-F/13460-R and 10214-F/13300-R. Nest PCR (PrimerSTAR MAX DNA Polymerase, R045A, TaKaRa) was performed using normal human DNA as a template. The second-round amplicons from the nest PCR were used as templates for amplification with pcMINI-*NTRK1*-KpnI-F/pcMINI-*NTRK1*-EcoRI-R and pcDNA3.1-*NTRK1*-KpnI-F/pcDNA3.1-*NTRK1*-EcoRI-R primers, respectively. This resulted in the fragments of pcMINI-*NTRK1*-wt (853 bp) and pcDNA3.1-*NTRK1*-wt (2,533 bp). For pcMINI-*NTRK1*-mut, the left and right halves of the fragment were separately amplified using pcMINI-*NTRK1*-KpnI-F/*NTRK1*-mut-R and *NTRK1*-mut-F/pcMINI-*NTRK1*-EcoRI-R primers, resulting in fragments of 386 bp and 498 bp, respectively. The left and right halves of the pcMINI-*NTRK1*-mut fragment were mixed in a 1:1 ratio and used as a template for amplification with pcMINI-*NTRK1*-KpnI-F and pcMINI-*NTRK1*-EcoRI-R primers, resulting in a fragment of 853 bp. The amplification steps for the pcDNA3.1-*NTRK1*-mut fragment were the same as for pcMINI-*NTRK1*-mut, but the primers used were pcDNA3.1-*NTRK1*-KpnI-F, *NTRK1*-mut-R, *NTRK1*-mut-F, and pcDNA3.1-*NTRK1*-EcoRI-R (the primers used for constructing the minigene vectors are shown in Supplementary Table S1). The sizes of the pcDNA3.1-*NTRK1*-mut left, right, and complete fragments were 166 bp, 2,227 bp, and 2,362 bp, respectively. The pcMINI-*NTRK1*-wt/mut and pcDNA3.1-*NTRK1*-wt/mut fragments were cloned into the pcMINI and pcDNA3.1 vectors, respectively, using KpnI/EcoRI restriction enzymes. Sequencing verification was performed to confirm the final constructs of the four vectors: pcMINI-*NTRK1*-wt/mut and pcDNA3.1-*NTRK1*-wt/mut.

### 2.5 Minigene splicing assay-transfection into eukaryotic cells

Human embryonic kidney cells (HEK293T; ATCC) and cervical cancer cells (HeLa; ATCC) were transfected with the four vectors separately (pcMINI-*NTRK1*-wt/mut and pcDNA3.1-*NTRK1*-wt/mut). The detailed transfection protocol could be referred to in a previous study (Miao et al., 2023). The cells were harvested 48 h post-transfection for further analyses.

### 2.6 Minigene splicing assay-reverse transcription-polymerase chain reaction (RT-PCR)

Total RNA from the HEK293T and HeLa cells transfected with the recombinant vectors was extracted using TRIzol reagent (RNAiso plus, Takara Bio Inc., Shiga, Japan). The concentration and purity of the extracted RNA samples were assessed. cDNA synthesis was performed using the HifairTM first Strand cDNA Synthesis Kit (11123ES70, YEASEN, Shanghai, China). PCR amplification was carried out using the flanking primers pcDNA3.1-F/pcDNA3.1-R (Supplementary Table S1) targeting the minigene vectors. The amplified gene transcribed bands were detected by agarose gel electrophoresis. The respective PCR bands were recovered and subjected to Sanger sequencing.

### 2.7 *In vitro* expression-construction of *NTRK1* expression vectors

A set of expression vectors, p3×Flag-CMV-7.1-wt/mut1/mut2, was constructed. Mut1 and mut2 corresponded to c.826_850del (p.Val276Ser fs*180) and c.838_850del (p.Val280Ser fs*180), respectively, representing two alternative splicing events. The EcoRI-wt-SalI fragment was amplified using a synthetic full-length *NTRK1* CDS as a template and primers p3×Flag-CMV-7.1-*NTRK1*-EcoRI-F/p3×Flag-CMV-7.1-*NTRK1*-SalI-R. The EcoRI and SalI double-digested fragment was then inserted into the p3×Flag-CMV-7.1 vector, resulting in the p3×Flag-CMV-7.1-wt vector, which was validated by sequencing. Using p3×Flag-CMV-7.1-wt as a template, mut1-1 and mut1-2 fragments were separately amplified with primers p3×Flag-CMV-7.1-*NTRK1*-EcoRI-F/*NTRK1*-mut1-R and *NTRK1*-mut1-F/p3×Flag-CMV-7.1-*NTRK1*-SalI-R, respectively. The obtained amplification products were mixed in a 1:1 ratio and subjected to a second-round PCR amplification using primers p3×Flag-CMV-7.1-*NTRK1*-EcoRI-F and p3×Flag-CMV-7.1-*NTRK1*-SalI-R, resulting in the EcoRI-mut1-SalI fragment. The mut1 fragment was double-digested with EcoRI and SalI, and ligated with the p3×Flag-CMV-7.1 vector, followed by sequencing validation, to generate the p3×Flag-CMV-7.1-mut1 vector. Similarly, the p3×Flag-CMV-7.1-mut2 vector was constructed using different primers (Supplementary Table S2) with the same procedure.

### 2.8 *In vitro* expression-transfection into eukaryotic cells

The constructed p3×Flag-CMV-7.1-wt/mut1/mut2 eukaryotic expression vectors were transfected into the HEK293T cell line using the previously described transfection method. After transfection, the cells were incubated for 48 h before collecting the samples for further experiments. The plasmid pCMV6-AC-GFP were co-transfected and subsequently detected to assess transfection efficiency.

### 2.9 *In vitro* expression-qPCR analysis

To synthesize cDNA, SuperMix for qPCR (gDNA digester plus) (11123ES70, YEASEN, Shanghai, China) kit was used following the previously described method. The cDNA samples were then subjected to qPCR analysis to determine the expression levels of *NTRK1* gene corresponding to p3×Flag-CMV-7.1-wt/mut1/mut2 vectors. The qPCR was performed using primers *NTRK1*-CMV-QPCR-F/*NTRK1*-CMV-QPCR-R (Supplementary Table S2).

### 2.10 *In vitro* expression-western blot analysis

Total protein from 293T cells was extracted using RIPA lysis buffer, and the protein concentration was determined using a BSA assay kit. The proteins were then denatured, and equal amounts of total protein were subjected to SDS-PAGE electrophoresis. Western blotting was performed using an anti-flag antibody, with GFP and GAPDH serving as internal references. This was done to detect the expression levels of wild-type and mutant target proteins of the p3×Flag-CMV-7.1 vectors.

### 2.11 Sanger sequencing

Sanger sequencing was utilized for validating the mutations identified by whole exome sequencing (WES). Simultaneously, it was also employed for verifying the constructs required for vector construction.

### 2.12 Statistical analysis

The intergroup data labeling was conducted using analysis of variance (ANOVA), and intergroup comparisons were tested using Dunnett’s test. *p* < 0.05 was considered statistically significant. Graphs were visualized and data were analyzed using GraphPad Prism software.

## 3 Results

### 3.1 Clinical features

**FIGURE 1:**
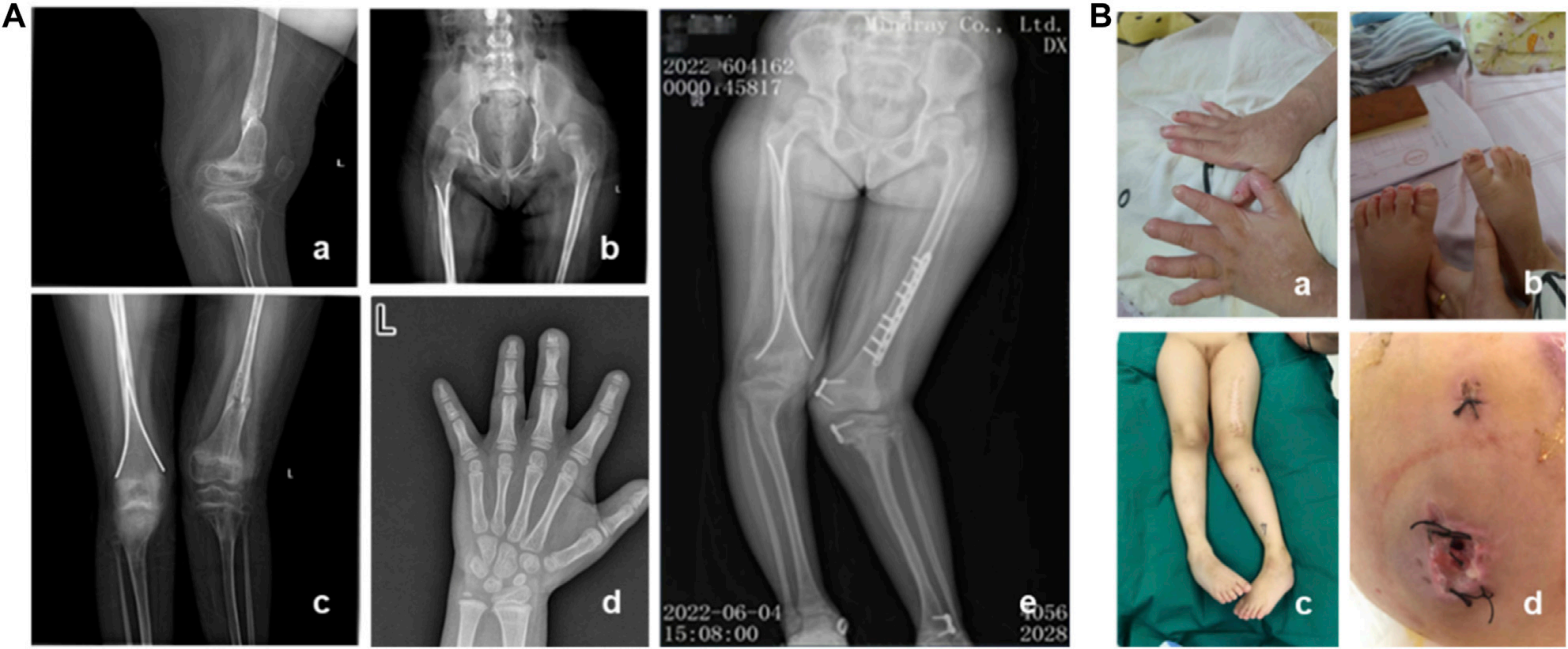
Clinical and imaging features of the patient. **(A)** Imaging characteristics of the patient. **(B)** Clinical features of the patient. a–b: Poor development of fingernails and toenails, along with scars from biting. c: Lower limb deformities and old surgical scars in the patient. d: One month after internal fixation, wound dehiscence occurred with subcutaneous purulent discharge.

A 7-year and 8-month-old female pediatric patient, who had previously undergone fracture surgery more than 1 year ago resulting in bilateral lower limb deformities, was admitted to our hospital. Over the course of the past 2 years, the patient experienced multiple fractures, initiating with a right lower limb fracture in July 2020 necessitating surgical intervention at an external medical facility. Subsequent to the surgery, a mild internal rotation deformity of the right knee manifested. In March 2021, a left lower limb fracture occurred, revealing pronounced external rotation of the left lower limb and inversion of the left ankle joint upon postoperative assessment. In January 2022, the patient underwent epiphyseal blocking procedures for the distal end of the left femur, proximal end of the left tibia, and distal end of the left tibia. Regrettably, postoperatively, deformities persisted in both lower limbs. Consequently, the patient underwent left femoral internal rotation osteotomy in our hospital in June 2022, with the potential extension of intramedullary nail fixation, removal of left femoral internal fixation, and removal of left tibial internal fixation. Unfortunately, postoperative complications included incision infection and challenges in wound healing. Clinical evaluation, coupled with narratives from the child and her parents, unveiled that she is the firstborn in the family, demonstrating slightly delayed growth and intellectual development, inadequate development of fingernails and toenails, and a history of recurrent fractures. The patient exhibits reduced sweating and diminished pain sensitivity since childhood. Additionally, she displays a proclivity for self-harm, frequently biting fingers and toenails, leading to multiple bite scars on the arms. The patient was clinically diagnosed as CIPA. Furthermore, irregular dental alignment and poor enamel development are evident. X-ray images revealed partial absence of the distal phalanges in digits 1-4 f the left hand. There was significant limb deformity, with the left knee showing an inward rotation of approximately 40° and the right knee showing an outward rotation of approximately 10°. The hip was dislocated, along with bilateral femoral abnormalities and old surgical scars. The left lower limb was approximately 2 cm longer than the contralateral side (Figure 1).

### 3.2 Pathogenicity analysis of *NTRK1*

**FIGURE 2:**
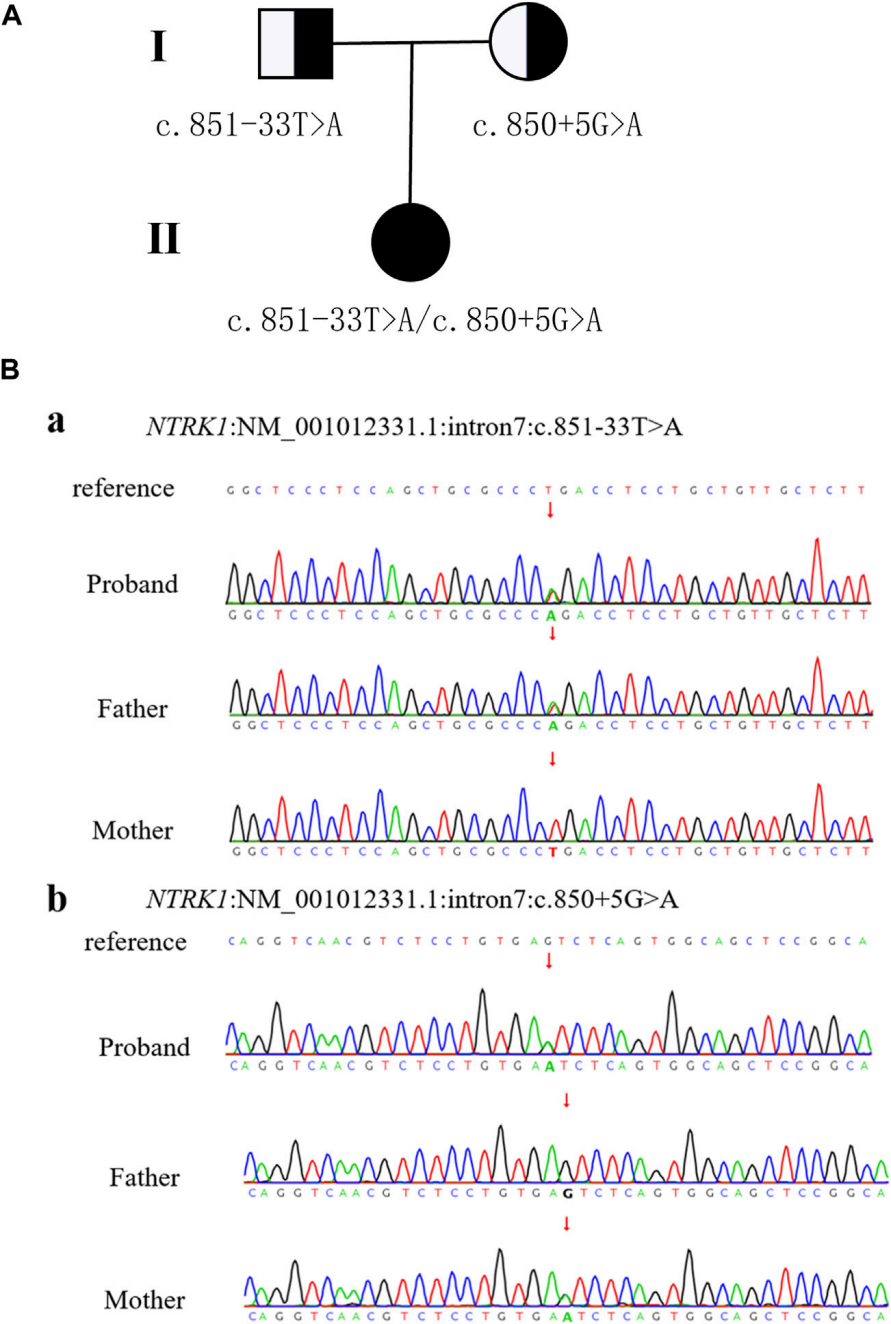
Patient pedigree and *NTRK1* variants. **(A)** Pedigree with *NTRK1* variants. **(B)** Sanger sequencing results of *NTRK1* in family members.

WES was performed on the proband as well as her parents. The results revealed two variants in *NTRK1* in the patient, which were confirmed by Sanger sequencing (Figure 2). The c.851-33T>A variant was inherited from her father and classified as a pathogenic based on the ACMG guidelines (PM3_VeryStrong + PS3 + PM2 + PP1_Moderate). The c.850 + 5G>A variant was inherited from her mother and was classified as a variant of uncertain significance (VUS) as it lacks population frequency data in the ExAC, 1000G, and gnomAD databases (PM2 + PM3 + PP3).

The c.850 + 5G>A variant was located in the fifth bp of intron 7, which was a "class II mutation region" known to affect splicing. SpliceAI, Varseak, and CBS tools were used to predict the impact of this variant on splicing. All three tools suggested that the variant would affect the splicing of the donor site (Table 1). SpliceAI predicted the loss of the original donor site after the variant (score 0.98), and the emergence of a new donor splice site near the variant (score 0.36). CBS predicted that the original donor site would also be lost after the variation. Varseak predicted a reduced score for the original donor site after the variation.

**TABLE 1:** *In silico* splicing analysis of the *NTRK1* variant.

Gene	Variant	SpliceAI		CBS		Varseak	
		Type	ΔScore	Type	Confidence	Type	Score (%)
*NTRK1*	c.850 + 5 G>A	Doner Loss	0.98	Before mutation	0.91	Doner Loss	36.56
		Doner Gain	0.36	After mutation	Nothing	Doner Gain	−63.69

### 3.3 The c.850 + 5G>A variant resulted in aberrant *NTRK1* splicing

**FIGURE 3:**
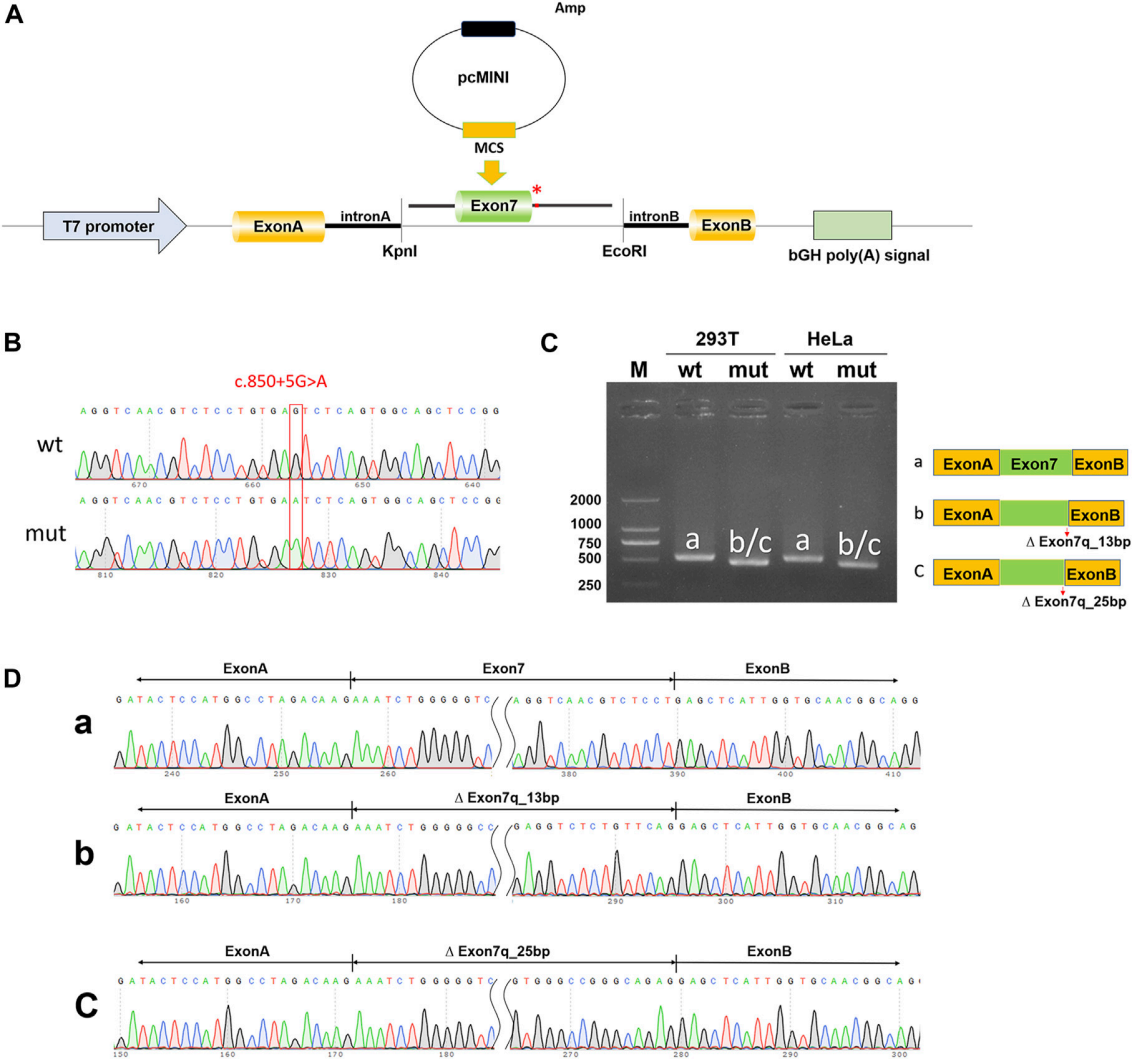
*In vitro* validation of pcMINI-*NTRK1*-wt/mut. **(A)** Schematic diagram of vector construction; red * indicates the variation site. **(B)** Sequencing results of the constructed vectors at the variation site. **(C)** Agarose gel electrophoresis and corresponding splicing diagram of the transcript analysis. **(D)** Sequencing results of the bands corresponding to **(C)**.

To validate the impact of the c.850 + 5G>A variant on *NTRK1* gene pre-mRNA splicing, pcMINI-*NTRK1*-wt/mut vectors were constructed (Figures 3A, B) and transfected into HeLa and 293T cell lines. In both cell lines, *NTRK1*-wt showed a single band (a-band) of the expected size (522bp) (Figure 3C). *NTRK1*-mut also showed a single band (Figure 3C). The splicing pattern of *NTRK1*-wt (a-band) was ExonA (192bp)-Exon7 (120bp)-ExonB (57bp) (Figure 3D). However, *NTRK1*-mut band represented two abnormal splicing patterns: b-band showed a 13bp deletion on the right side of Exon7, resulting in ExonA (192bp)-∆ Exon7 (120bp)-ExonB (57bp), while c-band showed a 25bp deletion on the right side of Exon7, resulting in ExonA (192bp)-∆ Exon7 (108bp)-ExonB (57bp) (Figure 3D).

**FIGURE 4:**
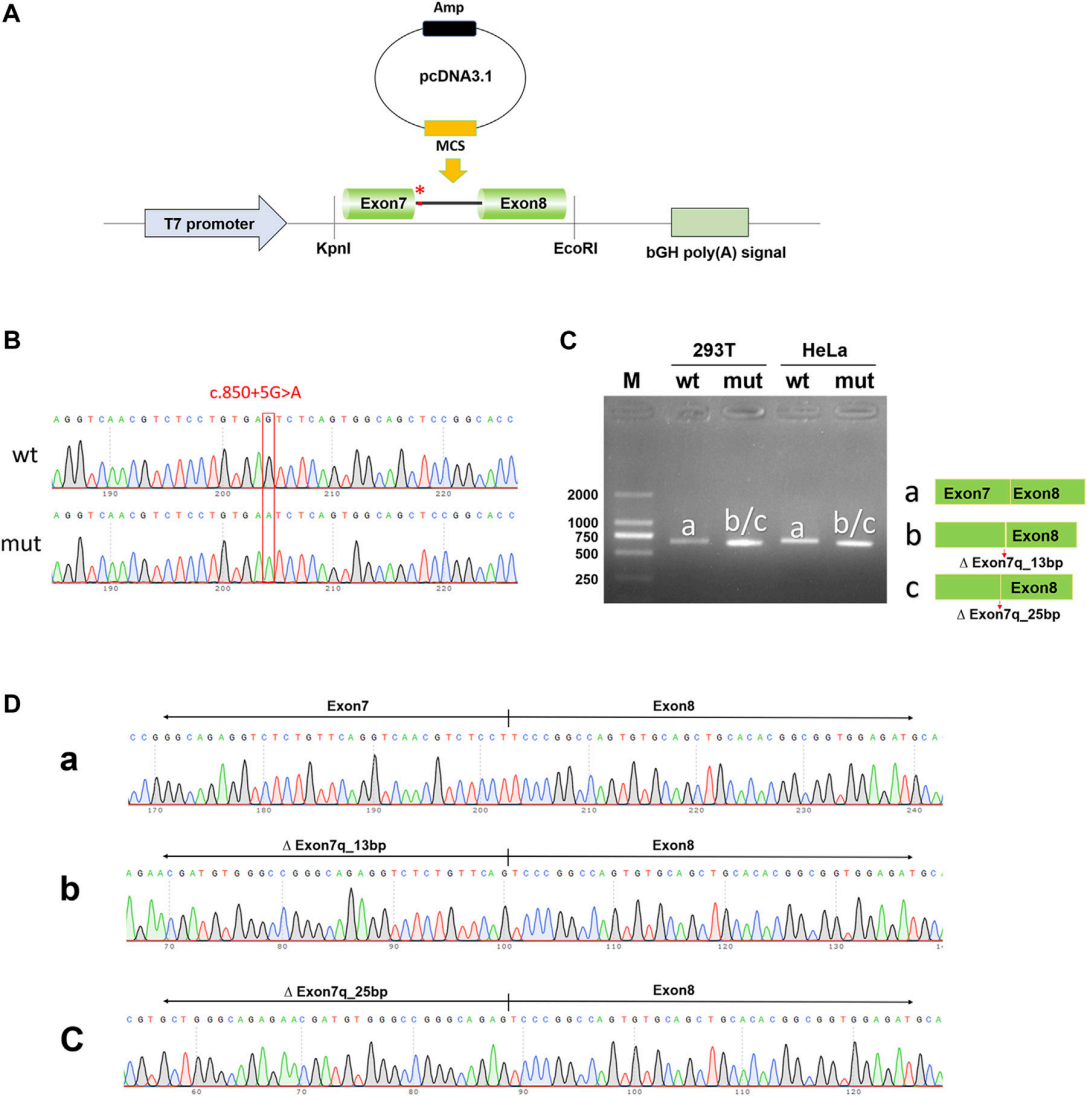
*In vitro* validation of pcDNA3.1-*NTRK1*-wt/mut. **(A)** Schematic diagram of vector construction; red * indicates the variation site. **(B)** Sequencing results of the constructed vectors at the variation site. **(C)** Agarose gel electrophoresis and corresponding splicing diagram of the transcript analysis. **(D)** Sequencing results of the bands corresponding to **(C)**.

To exclude the influence of the vector, pcDNA3.1-*NTRK1*-wt/mut vectors were also constructed (Figures 4A, B) and transfected into HeLa and 293T cell lines. The size of the *NTRK1*-wt (a-band) was consistent with the expected size (647bp) (Figure 4C), and the splicing pattern was Exon7 (133bp)-Exon8 (327bp) (Figure 4D). As expected, the *NTRK1*-mut band showed two abnormal splicing patterns (Figure 4D). b-band represented a 13bp deletion on the right side of Exon7, resulting in ∆ Exon7 (120bp)-Exon8 (327bp) splicing pattern (Figure 4D). c-band represented a 25bp deletion on the right side of Exon7, resulting in ∆ Exon7 (108bp)-Exon8 (327bp) splicing pattern (Figure 4D).

Therefore, the results from both the pcMINI and pcDNA3.1 minigene constructs demonstrated that the c.850 + 5G>A variant affected normal splicing of *NTRK1* mRNA. Both sets of vectors showed consistent results. The 13bp and 25bp deletions on the right side of Exon7 resulted in the production of premature termination codons (PTCs) in Exon11, ultimately leading to the production of truncated proteins of 458aa and 454aa, respectively, denoted as c.838_850del13bp (p.Val280Serfs*180) and c.826_850del25bp (p.Val276Serfs*180).

### 3.4 c.850 + 5G>A variant reduced *NTRK1* mRNA and protein expression

**FIGURE 5:**
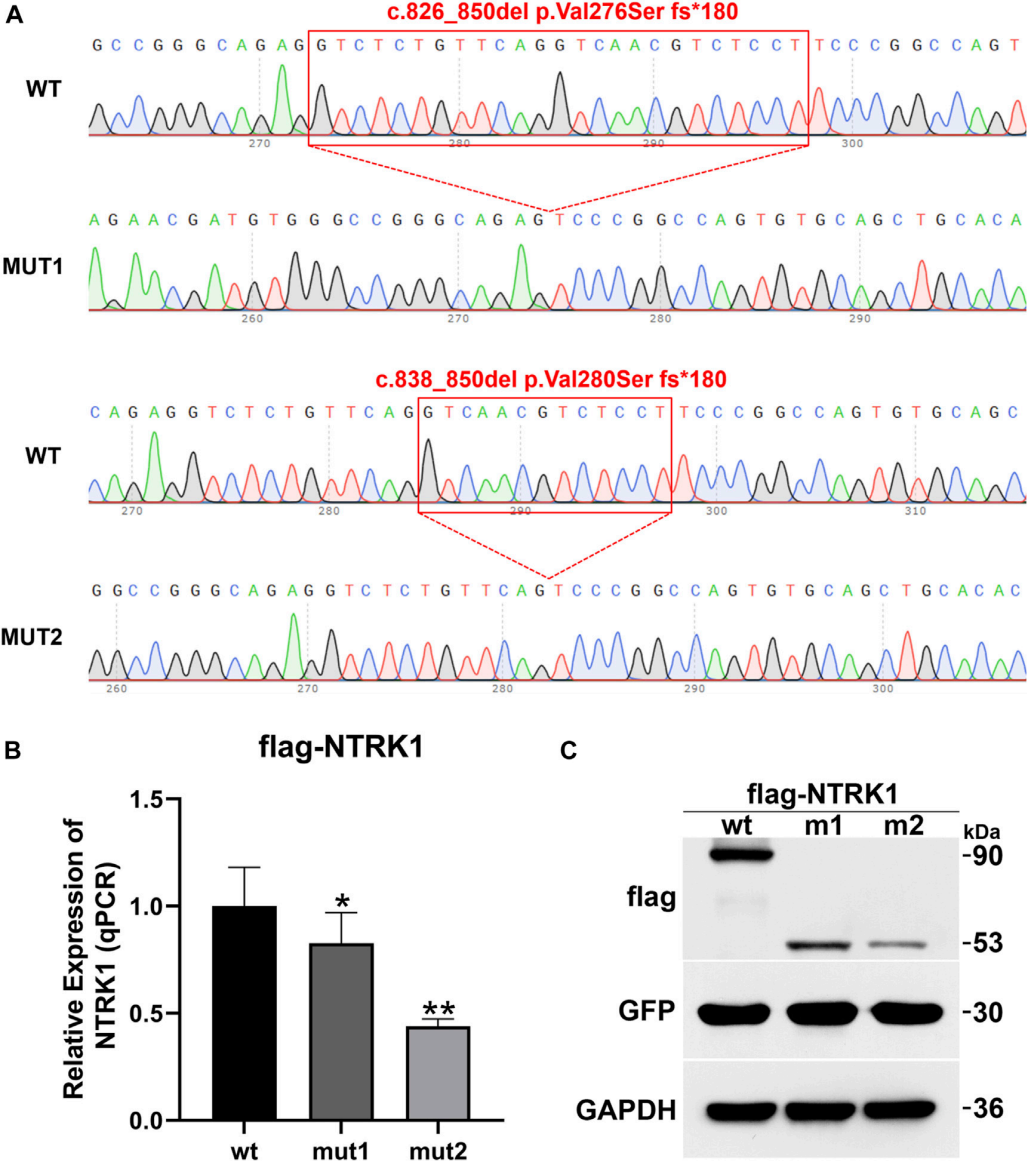
*In vitro* mRNA and protein expression results of *NTRK1*. **(A)** Sequencing results of the constructed p3×Flag-CMV-7.1-*NTRK1* wt/mut1/mut2 vectors (variation site). **(B)** Relative expression levels of *NTRK1* mutant and wild-type mRNA (qPCR). *: *p* values < 0.05, **: *p* values < 0.01. **(C)** Relative expression levels of *NTRK1* mutant and wild-type protein (WB).

To investigate the impact of the two aberrant splicing patterns on *NTRK1* expression, p3×Flag-CMV-7.1-wt/mut1/mut2 expression vectors were constructed for *in vitro* expression (Figure 5A). The expression levels of the wild-type and the two mutant transcripts were compared using qPCR. Compared to the wild-type control, the ex-pression of mut1 (c.826_850del,p.Val276Serfs*180) was reduced to 0.83, while the ex-pression of mut2 (c.838_850del,p.Val280Serfs*180) was reduced to 0.44 (Figure 5B).

Similarly, at the protein level, the wild-type protein was predicted to be 90 kDa. However, both mut1 and mut2 variants produced truncated proteins (mut1 predicted size: 53kDa, mut2 predicted size: 53 kDa), and their expression levels were also significantly lower compared to the wild-type (Figure 5C).

## 4 Discussion

In our study, we identified two *NTRK1* variants (c.851-33T>A and c.850 + 5G>A) in the affected girl of the family. The c.850 + 5G>A variant is novel, with no prior report, whereas the c.851-33T>A variant represents a known hotspot mutation at this locus of the *NTRK1* gene, associated with CIPA, expected to disrupt normal functionality, particularly prevalent in East Asian populations (Lee et al., 2009; Wang et al., 2017; Wang et al., 2018; Li et al., 2019; Zhao et al., 2020; Li L. et al., 2021; Yang et al., 2021). The c.851-33T>A variant was first identified and confirmed by Yuichi Miura et al. in Japanese CIPA patients in 2000. This variant leads to aberrant splicing of intron seven *in vitro*, resulting in the insertion of a 137 bp fragment (Miura et al., 2000). Additionally, c.851-33T>A variant was also found in two CIPA patients by Wen-bo Wang et al., with one of the patients being a girl (5 years old) who carried a homozygous c.851-33T>A variant. This patient experienced recurrent hyperthermia within the first year of life and exhibited typical symptoms of insensitivity to pain, anhidrosis, bone fractures, and self-mutilation. However, these symptoms gradually improved with age, presenting a milder CIPA phenotype (Wang et al., 2018). Among 21 Chinese CIPA patients reported by F. Zhao et al., 12 patients carried the c.851-33T>A variant. Among them, a boy (1 year and 7 months old) harboring a homozygous c.851-33T>A variant showed similar symptoms of insensitivity to pain, anhidrosis, bone fractures, recurrent hyperthermia, developmental delay, self-mutilation, and irritability. However, the phenotype of this patient was not completely consistent with that of the patients carrying a homozygous c.851-33T>A variant reported by Wen-bo Wang et al. This patient had intellectual disability and irritability, and it was unknown whether these symptoms improve with age (Zhao et al., 2020). Moreover, in another study, two patients (19 and 11 years old) carrying homozygous c.851-33T>A variants exhibited classic CIPA phenotypes, but the symptoms were not entirely consistent (Li et al., 2019). Patients with compound heterozygous variants involving the c.851-33T>A variant reported by F. Zhao et al. also showed significant clinical heterogeneity. Among them, the patient carrying the c.1736delT variant only showed symptoms of insensitivity to pain and anhidrosis, while the others presented with severe symptoms such as insensitivity to pain, anhidrosis, bone fractures, recurrent hyperthermia, developmental delay, self-mutilation, and irritability. The patient in our study exhibited typical CIPA symptoms, including insensitivity to pain, anhidrosis, self-mutilation, intellectual and growth retardation, and bone fractures. Furthermore, dental enamel dysplasia and lower limb dysplasia were also observed, which were also reported in one of the cases reported by Wen-bo Wang et al. (Wang et al., 2018). In this study, we further reviewed and summarized the clinical phenotypes of Chinese CIPA patients with different compound heterozygous combinations involving the *NTRK1* c.851-33T>A variant. The severity of the phenotype may be associated with different variant combinations, but further functional experiments are needed to investigate the specific impact of each type of variant on *NTRK1* protein function and its downstream effects (Table 2).

**TABLE 2:** The clinical manifestations of *NTRK1* gene c.851-33T>A variants in Chinese.

Patients	Gender	Age at last visit	Insensitivity to pain	Anhidrosis	Bone fractures	Recurrent fever	Mental retardation	Self-mutilation	Irascibility	Others	Zygote type	Allele origin	Region	Nucleotide (amino acid) change	References
1	M	6	Y	Y	Y	Y	N	Y	N		Het	F	I7	c.851-33T>A	Zhao et al. (2020)
												M	E5	c.474delG (p.W158Cfs*39)	
2	M	9	Y	Y	Y	Y	Y	Y	Y		Het	F	I7	c.851-33T>A	Zhao et al. (2020)
												M	E13	c.1784T>G(p.L595R)	
3	M	8	Y	Y	Y	Y	Y	Y	Y		Het	F	I7	c.851-33T>A	Zhao et al. (2020)
												M	E15	c.2066C>T(p.P689L)	
4	M	4	Y	Y	N	Y	Y	Y	Y		Het	M	E15	c.2066C>T(p.P689L)	Zhao et al. (2020)
												F	I7	c.851-33T>A	
5	M	1.58	Y	Y	Y	Y	Y	Y	Y		Hom	F/M	I7	c.851-33T>A	Zhao et al. (2020)
6	M	19	Y	Y	Y	Y	Y	Y	Y		Het	F	I7	c.851-33T>A	Zhao et al. (2020)
												M	E15	c.2122G>A (p.G708S)	
7	M	4.58	Y	Y	Y	Y	Y	Y	Y		Het	F	I7	c.851-33T>A	Zhao et al. (2020)
												M	E13	c.1750G>A (p.E584K)	
8	F	7	Y	Y	N	Y	Y	Y	Y		Het	F	I7	c.851-33T>A	Zhao et al. (2020)
												M	E15	c.2051C>T (p.P684L)	
9	F	UN	Y	Y	UN	UN	UN	UN	UN		Het	F	I7	c.851-33T>A	Zhao et al. (2020)
												M	E13	c.1736delT (p.L579Rfs *73)	
10	F	4.75	Y	Y	N	Y	Y	Y	Y		Het	F	I7	c.[851-798C>T; 851-794C>G]	Zhao et al. (2020)
												M	I7	c.851-33T>A	
11	F	0.58	Y	Y	N	Y	N/A	Y	N/A		Het	F	E5	c.447_450dupTCTG (p.H151Sfs *23)	Zhao et al. (2020)
												M	I7	c.851-33T>A	
12	M	9	Y	Y	Y	Y	Y	Y	Y		Het	F	E15	c.2066C>T (p.P689L)	Zhao et al. (2020)
												M	I7	c.851-33T>A	
13	M	15	Y	Y	Y	N/A	Y	N/A	N/A		Het	F	I7	c.851–33T > A	Yang et al. (2021)
												M	E16	c.2242C > T (p.R748W)	
14	M	2	Y	Y	N	Y	Y	Y	UN	Fall dawn、Severe infection	Het	F	I7	c.851-33T>A	Li et al. (2019)
15	M	11	Y	Y	Y	Y	Y	Y	UN	Slow wound healing、 Fall dawn、Severe infection	Het	M	I7	c.851-33T>A	Li et al. (2019)
16	M	3	Y	Y	Y	Y	Y	Y	UN	Fall dawn	Het	F	I7	c.851-33T>A	Li et al. (2019)
												M	E16	c.2197G>A (p.G714D)	
17	M	3	Y	Y	UN	Y	UN	UN	UN		Het	F	I7	c.851-33T>A	Li et al. (2019)
18	F	5	Y	Y	N	Y	UN	UN	UN		Het	F	I7	c.851-33T>A	Li et al. (2019)
19	M	5	Y	Y	N	Y	Y	Y	UN	Severe infection	Het	F	I7	c.851-33T>A	Li et al. (2019)
												M	E13	c.1736delT (p.L579Rfs *73)	
20	F	9	Y	Y	Y	Y	Y	Y	UN	Slow wound healing、Severe infection	Het	M	I7	c.851-33T>A	Li et al. (2019)
21	M	3	Y	Y	N	Y	Y	Y	UN	Fall dawn	Het	F	I7	c.851-33T>A	Li et al. (2019)
												M	I2	c.287+2dupT	
22	F	19	Y	Y	Y	Y	N	Y	UN		Hom	F/M	I7	c.851-33T>A	Li et al. (2019)
23	F	11	Y	Y	Y	Y	N	Y	UN	Fall dawn、Severe infection	Hom	F/M	I7	c.851-33T>A	Li et al. (2019)
24	M	0.42	Y	Y	N	Y	UN	Y	UN	Slow wound healing	Het	M	I7	c.851-33T>A	Li et al. (2019)
25	M	2	Y	Y	N	Y	Y	Y	UN	Fall dawn	Het	F	E8	c.963delG (p.L322Sfs^b^l48)	Li et al. (2019)
												M	I7	c.851-33T>A	
26	M	12	Y	Y	Y	Y	Y	Y	UN	Slow wound healing、 Fall dawn	Het	M	I7	c.851-33T>A	Li et al. (2019)
27	M	6	Y	Y	Y	Y	Y	Y	UN	Slow wound healing	Het	M	I7	c.851-33T>A	Li et al. (2019)
28	F	2	Y	Y	N	Y	Y	Y	UN	Fall dawn	Het	F	E8	c.1711_1721del (p.G571Rfs^b^10)	Li et al. (2019)
												M	I7	c.851-33T>A	
29	F	5	Y	Y	Y	Y	Y	Y	UN	Fall dawn	Het	F	I7	c.851-33T>A	Li et al. (2019)
												M	E6	c.632T>A (p.V211E)	
30	M	2	Y	Y	N	Y	Y	Y	UN	Fall dawn	Het	F	I7	c.851-33T>A	Li et al. (2019)
												M	E14	c.1805G>A (p.R602Q)	
31	M	2	Y	Y	N	Y	Y	Y	UN	Slow wound healing、 Fall dawn、Severe infection	Het	F	I7	c.851-33T>A	Li et al. (2019)
												M	E16	c.2066C>T (p.P689L)	
32	M	6	Y	Y	N	Y	Y	Y	UN	Slow wound healing、 Fall dawn、Severe infection	Het	F	I7	c.851-33T>A	Li et al. (2019)
												M	E16	c.2066C>T (p.P689L)	
33	F	7	Y	Y	Y	Y	Y	Y	UN	Fall dawn	Het	F	I7	c.851-33T>A	Li et al. (2019)
												M	I2	c.287+2dupT	
34	F	5	Y	Y	Y	Y	Y	Y	UN	Slow wound healing	Het	F	E5	c.446_447insTCTG(p.P149fs)	Li et al. (2019)
												M	I7	c.851-33T>A	
35	M	2	Y	Y	N	Y	Y	Y	UN	Fall dawn、Severe infection	Het	F	I7	c.851-33T>A	Li et al. (2019)
												M	I2	c.287+2dupT	
36	M	5	Y	Y	UN	Y	UN	UN	UN	Slow wound healing	Het	M	I7	c.851-33T>A	Li et al. (2019)
37	F	3	Y	Y	N	Y	Y	Y	UN	Slow wound healing、 Fall dawn	Het	M	I7	c.851-33T>A	Li et al. (2019)
38			Y	Y	Y	Y	N	Y	N		Het	M	I7	c.851-33T>A	Geng et al. (2018)
												F	I7	c.[851-798C>T; 851-794C>G]	
39			Y	Y	Y	Y	Y	Y	N		Het	F	I7	c.851-33T>A	Geng et al. (2018)
												M	E13	c.1736del(p.L579Rfs*73)	
40			Y	Y	Y	Y	Y	Y	Y		Het	F	I7	c.851-33T>A	Geng et al. (2018)
												M	E13	c.1750G>A(p.E584K)	
41			Y	Y	Y	Y	Y	Y	N	Osteomyelitis	Het	M	I2	c.287+2dupT	Geng et al. (2018)
												F	I7	c.851-33T>A	
42			Y	Y	Y	Y	Y	Y	Y		Hom	F/M	I7	c.851-33T>A	Geng et al. (2018)
43			Y	Y	Y	Y	Y	Y	N		Hom	F/M	I7	c.851-33T>A	Geng et al. (2018)
44			Y	Y	Y	Y	Y	Y	Y		Hom	F/M	I7	c.851-33T>A	Geng et al. (2018)
45			Y	Y	N	Y	Y	Y	Y		Het	M	I7	c.851-33T>A	Geng et al. (2018)
												F	E13	c.1786C>T(p.R596*)	
46			Y	Y	N	Y	Y	Y	Y	Osteomyelitis	Het	M	I7	c.851-33T>A	Geng et al. (2018)
												F	E13	c.1885G>C(p.A629P)	
47			Y	Y	N	Y	Y	Y	Y		Het	M	I7	c.851-33T>A	Geng et al. (2018)
												F	E13	c.1711_1721del(p.G571Rfs*10)	
48			Y	Y	Y	Y	Y	Y	N		Het	M	I7	c.851-33T>A	Geng et al. (2018)
												F	E15	c.2162C > T(p.P721L)	
49			Y	Y	Y	Y	Y	Y	Y		Het	F	I7	c.851-33T>A	Geng et al. (2018)
												M	E8	c.963del(p.L322S fs*142)	
50	M	5.8	Y	Y	Y	Y	N	N	N		Het	F	I7	c.851-33T>A	Lv et al. (2017)
												M	E17	c.2281C>T (p.R761W)	
51	M	20	Y	Y	Y	Y	N	N	N		Het	F	E14	c.1652delA	Lv et al. (2017)
												M	I7	c.851-33T>A	
52	F	27	Y	Y	Y	Y	Y	Y	Y	Severe infection	Hom	F/M	I7	c.851-33T>A	Liu et al. (2018)
53	F	5	Y	Y	UN	Y	Y	Y	UN		Het	F	E12	c.1415delG; p.G472fs	Li et al. (2012)
												M	I7	c.851-33T>A	
54	M	20	Y	Y	Y	Y	Y	UN	UN		Het	UN	I7	c.851-33T>A	Guo et al. (2004)
55	M	18	Y	Y	Y	Y	Y	UN	UN		Het	UN	I7	c.851-33T>A	Guo et al. (2004)
56	F	5	Y	Y	Y	Y	UN	Y	UN		Hom	F/M	I7	c.851-33T>A	Wang et al. (2018)
57	F	18	Y	Y	Y	Y	Y	Y	UN		Het	F	I7	c.851-33T>A	Wang et al. (2018)
												M	E1	c.44G>A	
58	M	13	Y	Y	Y	Y	N	N	UN	Severe infection	Hom	UN	I7	c.851-33T>A	Li et al. (2021c)
59	M	1.25	Y	Y	Y	Y	Y	Y	UN		Het	M	E5-E7	g.6995_11999del(E5-7del)	Li et al. (2021a)
												F	I7	c.851-33T>A	
60	F	13	Y	Y	Y	Y	Y	Y	Y		Hom	F/M	I7	c.851-33T>A	Li et al. (2021b)
61	F	3.5	Y	Y	Y	Y	Y	Y	Y		Hom	F/M	I7	c.851-33T>A	Li et al. (2021b)
62	F	2.5	Y	Y	N	Y	N	Y	N	Dislocation of the right hip	Het	M	I7	c.851-33T>A	Li et al. (2021b)
												F	I6	c.717 + 2T>C	
63	F	3.5	Y	Y	N	Y	Y	Y	Y		Het	F	I7	c.851-33T>A	Li et al. (2021b)
												M	I10	c.1251 + 1G>A	
64	M	10	Y	Y	Y	Y	Y	Y	Y		Hom	F/M	I7	c.851-33T>A	Li et al. (2021b)
65	M	3	Y	Y	Y	Y	Y	Y	Y		Hom	F/M	I7	c.851-33T>A	Li et al. (2021b)
66	F	17	Y	Y	Y	Y	UN	Y	UN		Het	F	I7	c.851-33T>A	Nam et al. (2017)
												M	I14	IVS14 + 3A>T	
67	F	16	Y	Y	Y	Y	UN	Y	UN		Het	F	I7	c.851-33T>A	Nam et al. (2017)
												M	I14	IVS14 + 3A>T	
68	M	14	Y	Y	Y	Y	UN	Y	UN		Het	F	I7	c.851-33T>A	Nam et al. (2017)
												M	I14	IVS14 + 3A>T	
69	M	28	Y	Y	Y	N	UN	Y	UN		Hom	F/M	I7	c.851-33T>A	Nam et al. (2017)
70	M	5	Y	Y	N	Y	N	Y	UN	Severe infection	Het	F	I7	c.851-33T>A	Li et al. (2012)
												M	E12	c.1415delG(p.G472fs_c.1415delG)	
71	F	7.6	Y	Y	Y	Y	Y	Y	UN	Severe infection Fall dawn	Het	F	I7	c.851-33T>A	
												M	I7	c.850 + 5G>A	

Y, yes; N, no; Un, unknown; F, female; M, male; E, exon; I, intron.

Through minigene splicing experiments and *in vitro* expression, we demonstrated that the newly identified c.850 + 5G>A variant led to two aberrant splicing events in *NTRK1*, resulting in reduced mRNA. In most cases, the introduction of a premature stop codon initiates nonsense-mediated mRNA decay *in vivo*. However, in our particular case, an abnormal splicing event results in a 180 amino acid (540 bp) extension before encountering the new stop codon. This suggests the potential for certain transcripts to evade nonsense-mediated decay. The expression experiment revealed that, in this scenario, the truncated protein expression level remains low. The c.850 + 5G>A variant caused the deletion of 13 bp and 25 bp of exon seven of *NTRK1*. Exon seven and exon eight of *NTRK1* encoded the extracellular immunoglobulin-like domain, which was crucial for binding to NGF (Lv et al., 2017). The aberrant splicing of exon seven observed in the patient in this study might directly disrupt the immunoglobulin-like domain and therefore affected the binding of NGF to TrKA. NGF can bind to TrKA to form dimers and enter the cells through either classic or folding-dependent endocytosis. This leads to phosphorylation events (Y496, Y676, Y680, Y681, etc.) that activate downstream signaling pathways including MAPK and PI3K, resulting in cascade effects (Wiesmann and de Vos, 2001; Marlin and Li, 2015) Variants in the *NTRK1* can affect the signal transduction of NGF-TrKA, leading to apoptosis of NGF-dependent nociceptive primary sensory neurons during development, loss of unmyelinated and thinly myelinated peripheral nerve fibers, and absence of innervation of sweat gland by postganglionic neurons of the sympathetic ganglia, ultimately causing insensitivity to pain and anhidrosis (Indo, 2018). There were also studies suggesting that the NGF-TrkA signaling was involved in the vascularization and ossification of cartilage models during embryonic development, which was critical for weight-bearing skeletal elements in adulthood (Tomlinson et al., 2016). Research by Franco et al. had shown that *NTRK1* variants can induce misfolding and aggregation of TrkA, leading to dysfunctional protein and resulting in neurodegeneration and varying degrees of cytotoxicity, which might be associated with the intellectual developmental delay observed in CIPA patients (Franco et al., 2016). In the case of the girl in this study who carried compound heterozygous variants c.851-33T>A and c.850 + 5G>A, the formation of truncated TrkA proteins was likely the underlying mechanism through which these variants contribute to the observed phenotype, possibly through the previously mentioned pathways.

## 5 Conclusion

In a CIPA pedigree, we identified a known East Asian hotspot splicing variant, c.851-33T>A, and a novel intronic variant, c.850 + 5G>A, in *NTRK1*. Analysis of the intronic variant, c.850 + 5G>A, revealed the generation of two truncated proteins, as well as a decrease in mRNA and protein expression levels, ultimately leading to the development of CIPA. This study expanded the variant spectrum of *NTRK1*, providing valuable insights for genetic diagnosis of CIPA and offering clues for the analysis of clinical phenotypes and underlying mechanisms associated with different *NTRK1* variants causing CIPA.

## Data Availability

The original contributions presented in the study are publicly available. This data can be found here: https://db.cngb.org/search/?q=CNP0005328.
