# Supplementary material for: A novel NTRK1 splice site variant causing congenital insensitivity to pain with anhidrosis in a Chinese family
Source: Front Genet. 2024 May 10;15:1345081. doi: 10.3389/fgene.2024.1345081 (PMC11116696; doi:10.3389/fgene.2024.1345081)
Supplement: Supplementary file 1 [file Table1.DOCX]

**Table S1: Minigene primer sequences**

| Primer | Sequence (5’-3’) |
| --- | --- |
| 10003-F | ctgtctaacctcacccatggt |
| 10214-F | gggatgcgtgacatctgttt |
| *NTRK1*-MUT-F | CAACGTCTCCTgtgaAtctcagtggcagctc |
| *NTRK1*-MUT-R | gagctgccactgagaTtcacAGGAGACGTTGA |
| 13300-R | ggcctcaaggaagaagatgc |
| 13460-R | gcgagaaggagactgcagca |
| pcDNA3.1-*NTRK1*-Kpnl-F | GCTTGGTACCATGAAATCTGGGGGTCTGCCATC |
| pcDNA3.1-*NTRK1*-EcoRI-R | TGCAGAATTCGTCAGGGATGGGGTCCTCGGGG |
| pcMINI-*NTRK1*-Kpnl-F | ggtaGGTACCcataaaataaaaaaaaaatg |
| pcMINI-*NTRK1*-EcoRI-R | TGCAGAATTCtttttgcacatttctatgta |
| pcDNA3.1-F | CTAGAGAACCCACTGCTTAC |
| pcDNA3.1-R(BGH-R) | TAGAAGGCACAGTCGAGG |

**Table S2: Construction of expression vectors and qPCR primers**

| Construction of expression vectors |  | Primer | Sequence (5’-3’) |
| --- | --- | --- | --- |
|  |  | *NTRK1*-mut1-F | GTGGGCCGGGCAGAGTCCCGGCCAGTGTGC |
|  |  | *NTRK1*-mut1-R | GCACACTGGCCGGGACTCTGCCCGGCCCAC |
|  |  | *NTRK1*-mut2-F | GAGGTCTCTGTTCAGTCCCGGCCAGTGTGC |
|  |  | *NTRK1*-mut2-R | GCACACTGGCCGGGACTGAACAGAGACCTC |
|  |  | p3×Flag-CMV-7.1-*NTRK1*-EcoRI-F | ccgcgaattcgATGCTGCGAGGCGGACGGCG |
|  |  | p3×Flag-CMV-7.1-*NTRK1*-SalI-R | tagagtcgacCTAGCCCAGGACATCCAGGT |
| qPCR | | *NTRK1*-p3×Flag-CMV-7.1-qpcr-F | TCAACAAATGTGGACGGAGA |
|  |  | *NTRK1*-p3×Flag-CMV-7.1-qpcr-R | GTGGTGAACACAGGCATCAC |
