# Supplementary material for: A novel NTRK1 splice site variant causing congenital insensitivity to pain with anhidrosis in a Chinese family
Source: Front Genet. 2024 May 10;15:1345081. doi: 10.3389/fgene.2024.1345081 (PMC11116696; doi:10.3389/fgene.2024.1345081)
Supplement: Supplementary file 2 [file Table2.DOCX]

**The CARE Checklist**

**1. Title – The diagnosis or intervention of primary focus followed by the words “case report”.**

Line 1-2/Page 1: A novel NTRK1 splice site variant causing congenital insensitivity to pain with anhidrosis in a Chinese family

Interpretation: No need to add “case report” to the title because it is not a case report article type.

**2. Key Words– 2 to 5 key words that identify diagnoses or interventions in this case report (including "case report").**

Line 17/Page 1: Keywords: CIPA; NTRK1; minigene assay; mRNA splicing; novel splice mutation

Interpretation: No need to add “case report” to keywords because it is not a case report article type.

**3. Abstract – (structured or unstructured)**

**3a. Introduction – What is unique about this case and what does it add to the scientific literature?**

Line 33-42/Page 1-2:

Results: One hotspot variant c.851-33T>A (ClinVar ID: 21308) and a novel variant c.850+5G>A was inherited from her father and mother, respectively, identified in the affected individuals. The c.850+5G>A variant in NTRK1 resulted in two forms of aberrant mRNA splicing: 13bp deletion (c.838_850del13, p.Val280Ser fs180) and 25bp deletion (826_850del25, p.Val276Ser fs180) in exon 7, both leading to a translational termination at a premature stop codon and forming a C-terminal truncated protein. The expression of two abnormal splicing isoforms was decreased both in the level of mRNA and protein. Conclusion: In conclusion, this study elucidated the genetic cause of a patient with CIPA and identified a novel variant c.850+5G>A in NTRK1, which broadened the and enriched the NTRK1 mutation spectrum.

**3b. The patient’s main concerns and important clinical findings.**

Line 19-27/Page 1:

Background: Congenital insensitivity to pain with anhidrosis (CIPA, OMIM #256800), also known as hereditary sensory and autonomic neuropathy type Ⅳ (HSAN-IV), is a rare autosomal recessive disorder characterized by recurrent episodic fevers, anhidrosis, insensitivity to noxious stimuli, self-mutilating behavior and intellectual disability. CIPA can be caused by the variants in NTRK1 gene, which encodes a high-affinity tyrosine kinase receptor for nerve growth factor. To ascertain the hereditary cause of a patient with CIPA accompanied by the additional symptoms of mild growth retardation, prone to fracture, underdeveloped nails of fingers and toes, irregular tooth alignment, enamel hypoplasia, postoperative wound healing difficulty, hand and limb deformity, and dislocation of hip joint.

**3c. The primary diagnoses, interventions, and outcomes.**

**Primary diagnoses:**

Line 24/Page 1: a patient with CIPA.

**Interventions and outcomes:**

N/A.

**3d. Conclusion – What are one or more “take-away” lessons from this case report?**

N/A.

**4. Introduction– Briefly summarizes why this case is unique and may include medical literature references.**

Line 90-96/Page 3:

In this study, we performed whole exome sequencing (WES) analysis to investigate the genetic etiology of CIPA in the affected child and identified c.851-33T>A mutation and **a novel c.850+5G>A variant in *NTRK1***. Additionally, we utilized computational prediction tools and conducted minigene experiments as well as in vitro expression assays to assess the impact of novel variations on the CIPA phenotype. Our findings expanded the spectrum of NTRK1 variants and laid the groundwork for further functional studies of NTRK1. Moreover, our results provide valuable insights for the genetic diagnosis of CIPA patients.

**5. Patient Information**

**5a. De-identified patient specific information.**

Line 99/Page 3: A 7-year and 8-month-old girl.

**5b. Primary concerns and symptoms of the patient.**

Line 99-101/Page 3:

A 7-year and 8-month-old girl, with a history of multiple fractures over the past two years, was admitted to our hospital due to lower limb deformity after fracture surgery more than one year ago. Despite self-reports and physical examinations, the cause could not be determined.

Additionally, Line 214-224/Page 6 in main manuscript described other symptoms of the patient.

**5c. Medical, family, and psychosocial history including relevant genetic information.**

**Medical history:**

Line 201-210/Page 6:

A 7-year and 8-month-old female pediatric patient, who had previously undergone fracture surgery more than one year ago resulting in bilateral lower limb deformities, was admitted to our hospital. Over the course of the past two years, the patient experienced multiple fractures, initiating with a right lower limb fracture in July 2020 necessitating surgical intervention at an external medical facility. Subsequent to the surgery, a mild internal rotation deformity of the right knee manifested. In March 2021, a left lower limb fracture occurred, revealing pronounced external rotation of the left lower limb and inversion of the left ankle joint upon postoperative assessment. In January 2022, the patient underwent epiphyseal blocking procedures for the distal end of the left femur, proximal end of the left tibia, and distal end of the left tibia. Regrettably, postoperatively, deformities persisted in both lower limbs.

**Family and psychosocial history**:

N/A. There is no family and psychosocial history.

**Genetic information:**

Line 227-231/Page 6:

The c.851-33T>A variant (Variation ID: 21308) was inherited from her father and classified as a pathogenic based on the ACMG guidelines (PM3_VeryStrong + PS3 + PM2 + PP1_Moderate). The c.850+5G>A variant was inherited from her mother and was classified as a variant of uncertain significance (VUS) as it lacks population frequency data in the ExAC, 1000G, and gnomAD databases (PM2 + PM3 + PP3).

**5d. Relevant past interventions and their outcomes.**

Line 201-210/Page 6: see the same content in **medical history** of **5c** as above.

**6. Clinical Findings– Describe significant physical examination (PE) and important clinical findings.**

Line 215-224/Page 6:

Slightly delayed growth and intellectual development, inadequate development of fingernails and toenails, and a history of recurrent fractures. The patient exhibits reduced sweating and diminished pain sensitivity since childhood. Additionally, she displays a proclivity for self-harm, frequently biting fingers and toenails, leading to multiple bite scars on the arms. The patient was clinically diagnosed as CIPA. Furthermore, irregular dental alignment and poor enamel development are evident. X-ray images revealed partial absence of the distal phalanges in digits 1-4 f the left hand. There was significant limb deformity, with the left knee showing an inward rotation of approximately 40° and the right knee showing an outward rotation of approximately 10°. The hip was dislocated, along with bilateral femoral abnormalities and old surgical scars. The left lower limb was approximately 2 cm longer than the contralateral side (Figure 1).

**7. Timeline – Historical and current information from this episode of care organized as a timeline (figure or table).**

Line 201-213/Page 6:

A 7-year and 8-month-old female pediatric patient, who had previously undergone fracture surgery more than one year ago resulting in bilateral lower limb deformities, was admitted to our hospital. Over the course of the past two years, the patient experienced multiple fractures, initiating with a right lower limb fracture in July 2020 necessitating surgical intervention at an external medical facility. Subsequent to the surgery, a mild internal rotation deformity of the right knee manifested. In March 2021, a left lower limb fracture occurred, revealing pronounced external rotation of the left lower limb and inversion of the left ankle joint upon postoperative assessment. In January 2022, the patient underwent epiphyseal blocking procedures for the distal end of the left femur, proximal end of the left tibia, and distal end of the left tibia. Regrettably, postoperatively, deformities persisted in both lower limbs. 这里加时间Consequently, the patient underwent left femoral internal rotation osteotomy in our hospital, with the potential extension of intramedullary nail fixation, removal of left femoral internal fixation, and removal of left tibial internal fixation. Unfortunately, postoperative complications included incision infection and challenges in wound healing.

**Summarize below**

| **Date** | **Cause** | **Treatment** | **Outcome** |
| --- | --- | --- | --- |
| 2020 July | Multiple fractures. | Surgical intervention. | A mild internal rotation deformity of the right knee manifested. |
| 2021 March | Left lower limb fracture | Surgical intervention. | Pronounced external rotation of the left lower limb and inversion of the left ankle joint. |
| 2022 January | - | Epiphyseal blocking procedures for the distal end of the left femur, proximal end of the left tibia, and distal end of the left tibia. | Deformities persisted in both lower limbs. |
| 2022 June | - | Left femoral internal rotation osteotomy. | postoperative complications included incision infection |

**8. Diagnostic Assessment**

**8a. Diagnostic methods (PE, laboratory testing, imaging, surveys).**

Line 213-218/Page 6:

Clinical evaluation, coupled with narratives from the child and her parents, unveiled that she is the firstborn in the family, demonstrating slightly delayed growth and intellectual development, inadequate development of fingernails and toenails, and a history of recurrent fractures. The patient exhibits reduced sweating and diminished pain sensitivity since childhood. Additionally, she displays a proclivity for self-harm, frequently biting fingers and toenails, leading to multiple bite scars on the arms.

**8b. Diagnostic challenges.**

N/A.

**8c. Diagnosis (including other diagnoses considered).**

Line 219-220/Page 6:

The patient was clinically diagnosed as CIPA.

**8d. Prognostic characteristics when applicable.**

N/A.

**9. Therapeutic Intervention**

**9a. Types of therapeutic intervention (pharmacologic, surgical, preventive).**

The patient accepted surgical intervention.

Line 210-213/Page 6:

Consequently, the patient underwent left femoral internal rotation osteotomy in our hospital, with the potential extension of intramedullary nail fixation, removal of left femoral internal fixation, and removal of left tibial internal fixation.

**9b. Administration of therapeutic intervention (dosage, strength, duration).**

N/A.

The surgery that patient accepted was not involved in dosage, strength and duration.

9c. Changes in therapeutic interventions with explanations.

N/A.

**10. Follow-up and Outcomes**

**10a. Clinician- and patient-assessed outcomes if available.**

Line 212-217/Page 6:

Unfortunately, postoperative complications included incision infection and challenges in wound healing. Clinical evaluation, coupled with narratives from the child and her parents, unveiled that she is the firstborn in the family, demonstrating slightly delayed growth and intellectual development, inadequate development of fingernails and toenails, and a history of recurrent fractures. The patient exhibits reduced sweating and diminished pain sensitivity since childhood.

**10b. Important follow-up diagnostic and other test results.**

Line 218-224/Page 6:

Additionally, she displays a proclivity for self-harm, frequently biting fingers and toenails, leading to multiple bite scars on the arms. Furthermore, irregular dental alignment and poor enamel development are evident. X-ray images revealed partial absence of the distal phalanges in digits 1-4 f the left hand. There was significant limb deformity, with the left knee showing an inward rotation of approximately 40° and the right knee showing an outward rotation of approximately 10°. The hip was dislocated, along with bilateral femoral abnormalities and old surgical scars. The left lower limb was approximately 2 cm longer than the contralateral side (Figure 1).

**10c. Intervention adherence and tolerability. (How was this assessed?)**

N/A.

**10d. Adverse and unanticipated events.**

Line 212-213/Page 6:

Unfortunately, postoperative complications included incision infection and challenges in wound healing.

**11. Discussion**

**11a.** **Strengths and limitations in your approach to this case.**

Line 274-277/Page 7:

In our study, we identified two *NTRK1* variants (c.851-33T>A and c.850+5G>A) in the affected girl of the family. The c.850+5G>A variant is novel, with no prior report, whereas the c.851-33T>A variant represents a known hotspot mutation at this locus of the *NTRK1* gene, associated with CIPA, expected to disrupt normal functionality, particularly prevalent in East Asian populations.

Line 308-315/Page 8:

Through minigene splicing experiments and in vitro expression, we demonstrated that the newly identified c.850+5G>A variant led to two aberrant splicing events in *NTRK1*, resulting in reduced mRNA. In most cases, the introduction of a premature stop codon initiates nonsense-mediated mRNA decay in vivo. However, in our particular case, an abnormal splicing event results in a 180 amino acid (540 bp) extension before encountering the new stop codon. This suggests the potential for certain transcripts to evade nonsense-mediated decay. The expression experiment revealed that, in this scenario, the truncated protein expression level remains low. The c.850+5G>A variant caused the deletion of 13 bp and 25 bp of exon 7 of *NTRK1*.

**11b. Discussion of the relevant medical literature.**

Please see Line 279-303/Page 7-8 in main manuscript as relevant text is long.

**11c. The rationale for your conclusions.**

Please see Line 310-334/Page 8 in main manuscript as relevant text is long.

**11d. The primary “take-away” lessons from this case report (without references) in a one paragraph conclusion.**

Line 331-334/Page 8:

In the case of the girl in this study who carried compound heterozygous variants c.851-33T>A and c.850+5G>A, the formation of truncated TrkA proteins was likely the underlying mechanism through which these variants contribute to the observed phenotype, possibly through the previously mentioned pathways.

**12. Patient Perspective – The patient should share their perspective on the treatment(s) they received.**

N/A.

**13. Informed Consent – The patient should give informed consent. (If not, explain)**

Line 368-371/Page 9:

All people included in this subject have provided written informed consents. The authors affirm that human research participants provided informed consent for publication of the images in Figure(s) 1A and 1B.
